# Optimizing SARS-CoV-2 vaccine responses in kidney transplant recipients: an urgent need

**DOI:** 10.1128/spectrum.00004-24

**Published:** 2024-05-15

**Authors:** Yi-Ling Cheng, Shen-Shin Chang, Chiao-Hsuan Chao, Po-Ta Chen, Ya-Lan Lin, Guan-Da Syu, Nan-Yao Lee, Po-Lin Chen, Wen-Chien Ko, Tzong-Shiann Ho

**Affiliations:** 1 Department of Pediatrics, National Cheng Kung University Hospital, College of Medicine, National Cheng Kung University, Tainan, Taiwan; 2 Department of Surgery, National Cheng Kung University Hospital, College of Medicine, National Cheng Kung University, Tainan, Taiwan; 3 Department of Medical Laboratory and Regenerative Medicine, MacKay Medical College, New Taipei, Taiwan; 4 Department of Internal Medicine, National Cheng Kung University Hospital, College of Medicine, National Cheng Kung University, Tainan, Taiwan; 5 Department of Biotechnology and Bioindustry Sciences, National Cheng Kung University, Tainan, Taiwan; 6 Medical Device Innovation Center, National Cheng Kung University, Tainan, Taiwan; 7 Department of Pediatrics, National Cheng Kung University Hospital Dou-Liou Branch, College of Medicine, National Cheng Kung University, Yunlin, Taiwan; 8 Center of Infectious Disease and Signaling Research, National Cheng Kung University, Tainan, Taiwan; National University of Singapore, Singapore, Singapore

**Keywords:** COVID-19, SARS-CoV-2, kidney transplanted recipient, vaccination, serological response, T cell response, cytokines, variant spike peptide pools

## Abstract

Kidney transplant recipients (KTRs) have been identified as a population at increased risk for severe SARS-CoV-2 infection outcomes. This study focused on understanding the immune response of KTRs post-vaccination, specifically examining both serological and cellular responses to the SARS-CoV-2 vaccine. Thirteen individuals, including seven KTRs and six healthy donors, were evaluated for antibody levels and T cell responses post-vaccination. The study revealed that KTRs had significantly lower serological responses, including reduced anti-receptor binding domain (RBD) binding antibodies and neutralizing antibodies against the Wuhan, Delta, and Omicron BA.2 strains. Additionally, KTRs demonstrated weaker CD8 T cell cytotoxic responses and lower Th1 cytokine secretion, particularly IFN-γ, after stimulation with variant spike peptide pools. These findings highlight the compromised immunity in KTRs post-vaccination and underscore the need for tailored strategies to bolster immune responses in this vulnerable group. Further investigations are warranted into the mechanisms underlying reduced vaccine efficacy in KTRs and potential therapeutic interventions.

**IMPORTANCE:**

Some studies have revealed that KTRs had lower serological response against SARS-CoV-2 than healthy people. Nevertheless, limited studies investigate the cellular response against SARS-CoV-2 in KTRs receiving SARS-CoV-2 vaccines. Here, we found that KTRs have lower serological and cellular responses. Moreover, we found that KTRs had a significantly lower IFN-γ secretion than healthy individuals when their PBMCs were stimulated with SARS-CoV-2 spike peptide pools. Thus, our findings suggested that additional strategies are needed to enhance KTR immunity triggered by the vaccine.

## INTRODUCTION

Severe acute respiratory syndrome coronavirus 2 (SARS-CoV-2) spreads rapidly worldwide and caused a global coronavirus disease 2019 (COVID-19) pandemic in December 2019. SARS-CoV-2 uses the receptor binding domain (RBD) on its spike protein to bind to the ACE2 receptor on the surface of host cells, enabling it to enter and infect cells (1, 2). Although most COVID-19 cases have mild symptoms, individuals with underlying comorbidities are at a higher risk of experiencing severe and life-threatening complications (3, 4). The administration of vaccines against SARS-CoV-2 not only reduced transmission of the virus but also mitigated the severity of illness and reduced mortality rates among those infected (5). Taiwan is facing an aging population structure and the prevalence of comorbidities such as cardiovascular and metabolic diseases, as well as chronic kidney disease. According to the statistics from the Taiwan Society of Nephrology, Taiwan has the world's highest incidence rate of end-stage kidney disease, with 476 cases per million, and the highest prevalence rate of 3,317 cases per million. Kidney transplantation surgery, with the aid of immunosuppressive agents, can significantly prolong life expectancy and improve the quality of life of end-stage kidney disease patients compared to dialysis. However, a previous study reported that both humoral and cellular immunities elicited by SARS-CoV-2 vaccines are significantly reduced in kidney transplant recipients (KTRs) compared to dialysis patients (6). Moreover, several studies have found that KTRs have a higher mortality rate compared to non-transplant patients; even after receiving two doses of mRNA COVID-19 vaccine, KTRs still experienced hospitalization in approximately 57.6% of cases and a mortality rate of 9.1% (7). These studies emphasize the need for ongoing vigilance and proactive management of KTRs during the COVID-19 pandemic (8, 9).

In addition to B cells for producing specific antibodies, T cells are essential in regulating immune responses and clearing intracellular and extracellular pathogens. Specifically, CD4 T cells can promote the activation of B cells and enhance the production of antigen-specific antibodies (10, 11). However, fewer studies have examined the cellular immune response (known as T cell responses) of KTRs to vaccination compared to those that have evaluated the antibody response in individuals without prior infection (12). In this study, we would like to assess the cellular immune response triggered by SARS-CoV-2 vaccination using the activation-induced marker (AIM) assay in KTRs (13–16). To achieve this aim, fresh isolated PBMCs from KTRs or healthy donors were stimulated with synthesized peptide pools of SARS-CoV-2 spike proteins, including those of the Wuhan, Delta, and Omicron BA.2 variants. The cytotoxic T cell response was evaluated by detecting activation markers (CD69, CD134, and CD137) on the surface of CD4 and CD8 T cells after 20 hours of peptide stimulation. The supernatants of peptide pools stimulated PBMCs, which were also collected to detect specific cytokine secretion, which helped to identify the polarization of CD4 T cells triggered by vaccination in KTRs in greater detail.

## MATERIALS AND METHODS

### Plasma collection and PBMC isolation

Lithium heparin tubes (Greiner Bio-One, Frickenhausen, Germany) were used to collect whole blood. After centrifugation for 5 minutes at 770 × g, plasma was collected and stored at −80°C, and antibody testing was completed within 6 months. PBMCs were isolated from the subjects within 2 hours after blood collection using density-gradient sedimentation, as described below. The whole blood was diluted with Roswell Park Memorial Institute (RPMI) 1640 medium (GeneDireX, Taiwan), layered over a 1:4 vol of Ficoll-Plaque (GE Healthcare, Chicago, IL), and centrifuged for 25 minutes at 1,200 × g with no brake. The PBMCs were collected, washed, and resuspended in an AIM medium (Thermo Fisher Scientific, Waltham, MA) containing 10% FBS (HyClone, Logan, UT) for AIM assay.

### SARS-CoV-2 peptide synthesis and pooling

Crude materials of SARS-CoV-2 Wuhan, B.1.617.2 (Delta), and B.1.1.529 (Omicron BA.2) peptides were synthesized (JPT, Berlin, Germany). The peptides were 15-mers overlapping by 11 amino acids and spanned the entire SARS-CoV-2 spike protein. For each antigen, peptide pools were resuspended in 500 µL of PBS with 30% glycerol (4 µL for stimulating 1 × 10^6^ cells).

### Detection of anti-RBD or anti-nucleocapsid-specific binding antibodies

Antibodies against SARS-CoV-2 nucleocapsid and SARS-CoV-2 RBD were detected using the Anti-SARS-CoV-2 and Anti-SARS-CoV-2S kits (Elecsys Corporation, USA), respectively, following the manufacturer's instructions. Briefly, plasma was incubated with a mixture of biotinylated and ruthenylated nucleocapsid or RBD antigens for 9 minutes. Streptavidin-coated microparticles were then added and set for an additional 9 minutes. The reagent mixture was finally measured with a photomultiplier (Cobas e602 module, Roche, USA). The cutoff index (COI) was used to determine the reactivity: COI < 1.0 was considered non-reactive, while COI ≥ 1.0 was considered reactive.

### Detection of neutralizing antibodies

To detect total neutralizing antibodies against SARS-CoV-2 (Wuhan, Delta, and Omicron BA.2) in the plasma samples, we used the cPass SARS-CoV-2 neutralization antibody detection kit (GenScript, Piscataway, NJ), also known as SARS-CoV-2 Surrogate Virus Neutralization Test (sVNT) kit. Briefly, S1-RBD conjugated with HRP (1:1,000 dilution with HRP dilution buffer) was pre-incubated with plasma (1:10 dilution with sample buffer) for 30 minutes at 37°C. Then, 100 µL of the mix samples was added to the ACE2 protein-coated plate for 15 minutes at 37°C. The wells were washed four times with wash buffer (20× wash buffer diluted with ddH2O, 260 µL/well), followed by the addition of TMB (100 µL/well). The plates were incubated for 15 minutes, and the reaction was stopped by adding 2N H_2_SO_4_ (50 µL/well). The absorbance was read at OD 450 nm using a VersaMax microplate reader (Molecular Devices, Sunnyvale, CA). The resulting OD values were further converted into inhibition rates (%) for analysis.

### Activation-induced cell marker (AIM) assay

PBMCs (1 × 10^6^ cells in 150 µL per well) were cultured in 96-well U-bottom plates with SARS-CoV-2 peptide pools, while negative solvent control was stimulated with an equal volume of PBS with 30% glycerol. After 20 hours of stimulation, supernatants were collected to detect specific cytokines using a cytometric bead array (CBA). Cells were washed with PBS containing 2% FBS and then stained with the following antibodies at 4°C: anti-CD3-FITC (1:10, clone HIT3a; BD Biosciences), anti-CD4-BV510 (1:20, clone SK3; BD Biosciences), anti-CD8-BV605 (1:20, clone SK1; BD Biosciences), anti-CD69-phycoerythrin (PE)-Cy7 (1:20, clone FN50; BD Biosciences), anti-CD134-PE-CF594 (1:20, clone ACT35; BD Biosciences), anti-CD137-BV421 (1:20, clone 4B4-1; BD Biosciences), and fixable viability stain 780 (1:1,000; BD Biosciences). After 30 minutes, cells were washed with PBS and fixed with 2% paraformaldehyde for 15 minutes at room temperature. Finally, cells were resuspended in PBS and acquired on a CytoFLEX S (Beckman Coulter, Brea, CA).

### Detection of cytokines by flow cytometry

Cytokines in cell culture supernatants from peptide stimulations were quantified using a cytometric bead array (CBA) human Th1/Th2 cytokine kit (BD Biosciences, Bergen, NJ). Briefly, the cell-cultured supernatants, all capture beads (which have been conjugated with specific antibodies against IL-2, IL-4, IL-5, IL-10, TNF, and IFN-γ), and detection reagent (phycoerythrin-conjugated antibodies) were mixed and incubated for 3 hours at room temperature. After washing with wash buffer, these sandwich complexes (capture beads + analyte + detection reagent) can be measured by flow cytometry on LSRFortessa (BD Biosciences). Cytokine levels were further analyzed using BD CellQuest software.

### Statistical analysis

The data were analyzed using GraphPad Prism version 5.0 (GraphPad Software Inc., CA) and presented as means ± standard deviations (SDs). We used the student's *t*-test to compare differences between the two groups, with *P* values < 0.05 considered statistically significant.

## RESULTS

### Characteristics of donor cohorts

To evaluate the serological and cellular responses of KTRs to SARS-CoV-2 vaccination, we included 13 participants in this study: seven KTRs and six healthy donors. All the participants had received SARS-CoV-2 vaccination and had no prior infection (Fig. S1). Table 1 summarizes the characteristics of all participants, including age, gender, and the duration after the last dose of vaccination. Additional information regarding vaccine brand and administration dates for each dose can be found in Fig. S2.

**TABLE 1:** Participant characteristics^*a*^

Variable	Number (%)		*P* value
	Healthy donor *n* = 6	Kidney transplanted recipient (KTR) *n* = 7	
Age, mean ± SD	43.7 ± 16.5	48.1 ± 14.8	0.3166
Gender, female	4 (66.7)	1 (14.3)	0.0759
Duration after the last dose of vaccination, days, mean ± SD	136.7 ± 45.8	115.6 ± 57.8	0.4314
Previous CMV infection	0 (0)	0 (0)	
Comorbidity			
Hypertension	0 (0)	3 (42.8)	
Coronary artery disease	0 (0)	1 (14.3)	
Diabetes mellitus	1 (16.7)	1 (14.3)	
Malignancy	0 (0)	0 (0)	
Dyslipidemia	0 (0)	1 (14.3)	
Autoimmune disease	0 (0)	0 (0)	

^*a*^ The characteristics, including age, gender, the duration after the last dose of vaccination, and comorbidity of all participants, were shown. SD: standard deviation, CMV: cytomegalovirus.

### KTRs had a lower serological response compared to healthy donors

**Fig 1:**
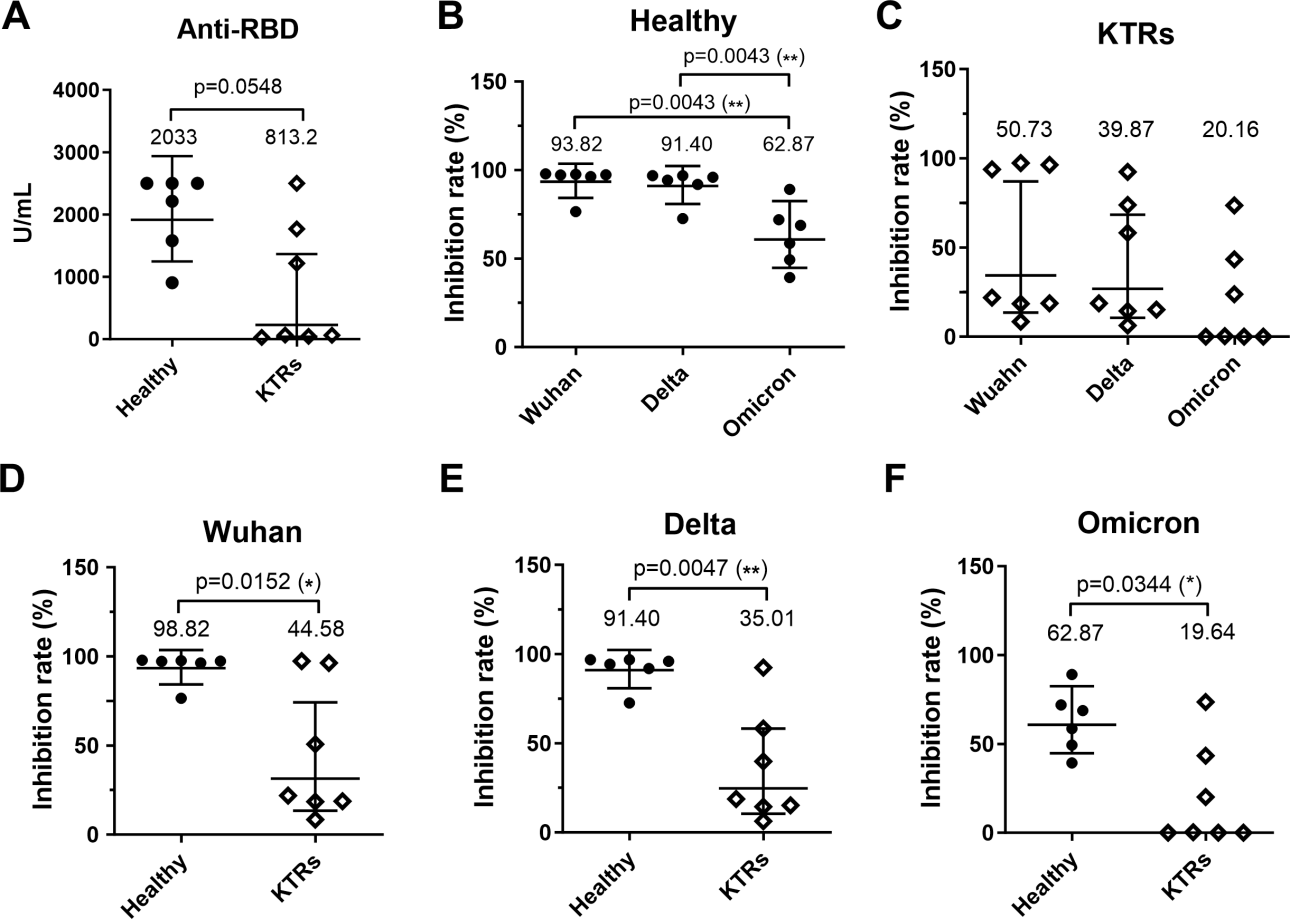
Kidney transplant recipients (KTRs) had a lower serological response than healthy donors. (**A**) The levels of anti-RBD binding antibodies were measured. The inhibition rates of neutralizing antibodies against Wuhan, Delta, and Omicron were compared in (**B**) healthy donors and (**C**) KTRs. The inhibition rates of neutralizing antibodies against (**D**) Wuhan, (**E**) Delta, and (**F**) Omicron between the healthy donor and the KTR groups.

To evaluate the serological response, the level of anti-RBD antibodies in the plasma of each participant was measured. In our results, the healthy donor group exhibited a mean value of 2,033 U/mL of anti-RBD antibodies in their plasma. In contrast, the KTRs group had a lower mean value of 813.2 U/mL of anti-RBD antibodies (Fig. 1A). Notably, four out of seven KTRs (57.1%) had detectable levels of anti-RBD antibodies below 70 U/mL. Next, the cPass SARS-CoV-2 neutralization antibody detection kit was utilized for determining the neutralizing antibody levels in each plasma sample against SARS-CoV-2 Wuhan, Delta, and Omicron BA.2. As shown in Fig. 1B, the healthy donor group had significantly higher inhibition rates for both cPass-Wuhan (93.82%) and cPass-Delta (91.4%) compared to cPass-Omicron BA.2 (62.87%). Similarly, the KTR groups exhibited the lowest inhibition rate in the plasma sample tested with cPass-Omicron BA.2 (20.16%) compared to the results of other tests (cPass-Wuhan, 50.73%; cPass-Delta, 39.87%) (Fig. 1C).

In the comparison of the KTR groups with the healthy donor groups, we observed the lower inhibition rates in all tests conducted for KTRs (cPass-Wuhan, *P* = 0.0152; cPass-Delta, *P* = 0.0047; cPass-Omicron BA.2, *P* = 0.0344) (Fig. 1D through E). Notably, four out of the seven KTRs (57.1%) exhibited inhibition rates lower than 30% when tested with cPass-Wuhan, cPass-Delta, and cPass-Omicron BA.2, suggesting that they did not possess neutralizing antibodies against the corresponding strains. Consistent with previous studies, our results indicate that KTRs had lower levels of anti-RBD binding and neutralizing antibodies compared to healthy donors (*P* = 0.0548), and a subset of KTRs (57.1%) exhibited weak serological responses to SARS-CoV-2 (Wuhan, Delta, and Omicron BA.2) (17, 18).

The correlation between cPASS inhibition values (%) and anti-RBD levels (BAU/mL, using the WHO reference panel) was also calculated, as shown in Fig. S5, to allow comparison with WHO units and studies worldwide.

### SARS-CoV-2-specific T cell response of KTRs was not significantly different from healthy donors

**Fig 2:**
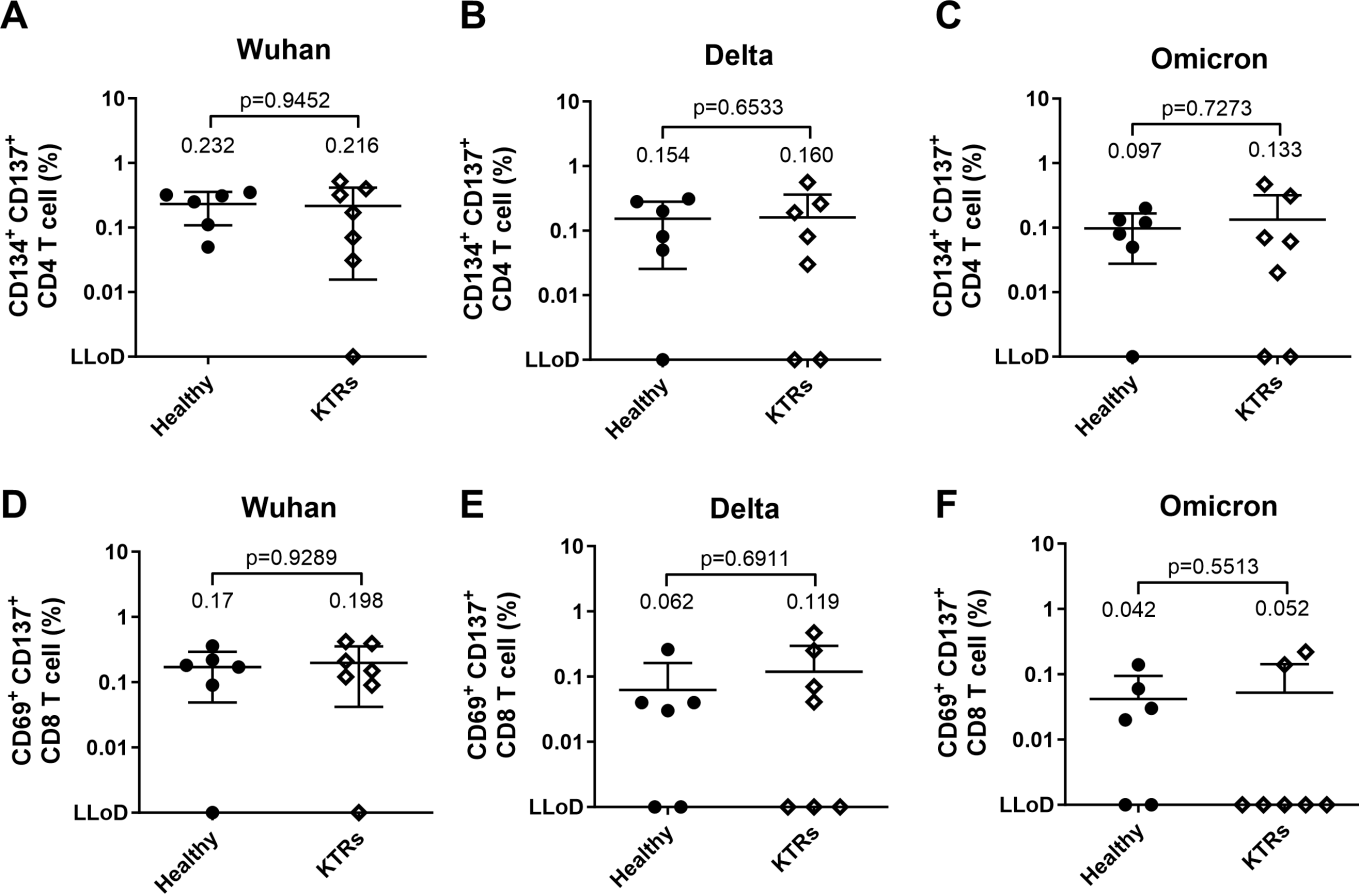
The T cell reactivity of kidney transplant recipients (KTRs) was not significantly different from that of healthy donors. SARS-CoV-2-specific CD4+ T cell responses against (**A**) Wuhan, (**B**) Delta, and (**C**) Omicron were measured after being stimulated with spike peptide pools. SARS-CoV-2-specific CD8+ T cell responses against (**D**) Wuhan, (**E**) Delta, and (**F**) Omicron were measured after being stimulated with spike peptide pools.

Given the lower serological response of KTRs compared to healthy donors, we aimed to investigate whether the KTRs could elicit T cell reactivity upon SARS-CoV-2 vaccination. To measure the T cell cytotoxic reactivity, the PBMCs from participants were stimulated with spike (Wuhan, Delta, or Omicron BA.2) peptide pools. The percentage of CD4 T cells (CD134+ CD137+) and that of CD8+ T cells (CD69+ CD137+) were analyzed using flow cytometry. The details of the gating strategy are provided in Fig. S3. We found no significant differences in CD4 or CD8 T cell reactivity between the healthy donor and the KTR groups (Fig. 2A through F). However, we observed that the CD4 and CD8 T cell reactivity was lower in PBMCs stimulated with Delta or Omicron BA.2 peptide pool compared to the Wuhan peptide pool (Fig. S4A through D). We also found that stimulation with variant (Delta and Omicron BA.2) peptide pools resulted in more participants with undetectable T cell reactivity than with Wuhan peptide pools (Fig. 4A through D). Notably, the CD8 T cell reactivity in KTRs was much lower with Omicron BA.2 peptide pool stimulation than with Wuhan peptide pool stimulation (*P* = 0.0693), as shown in Fig. S4D. These results suggested that the KTR groups showed weaker CD8 T cell cytotoxic responses against variant spike peptide pools, although with three vaccination doses.

### KTRs had lower production of Th1 cytokine and higher production of Th2 cytokine compared to healthy donors

**Fig 3:**
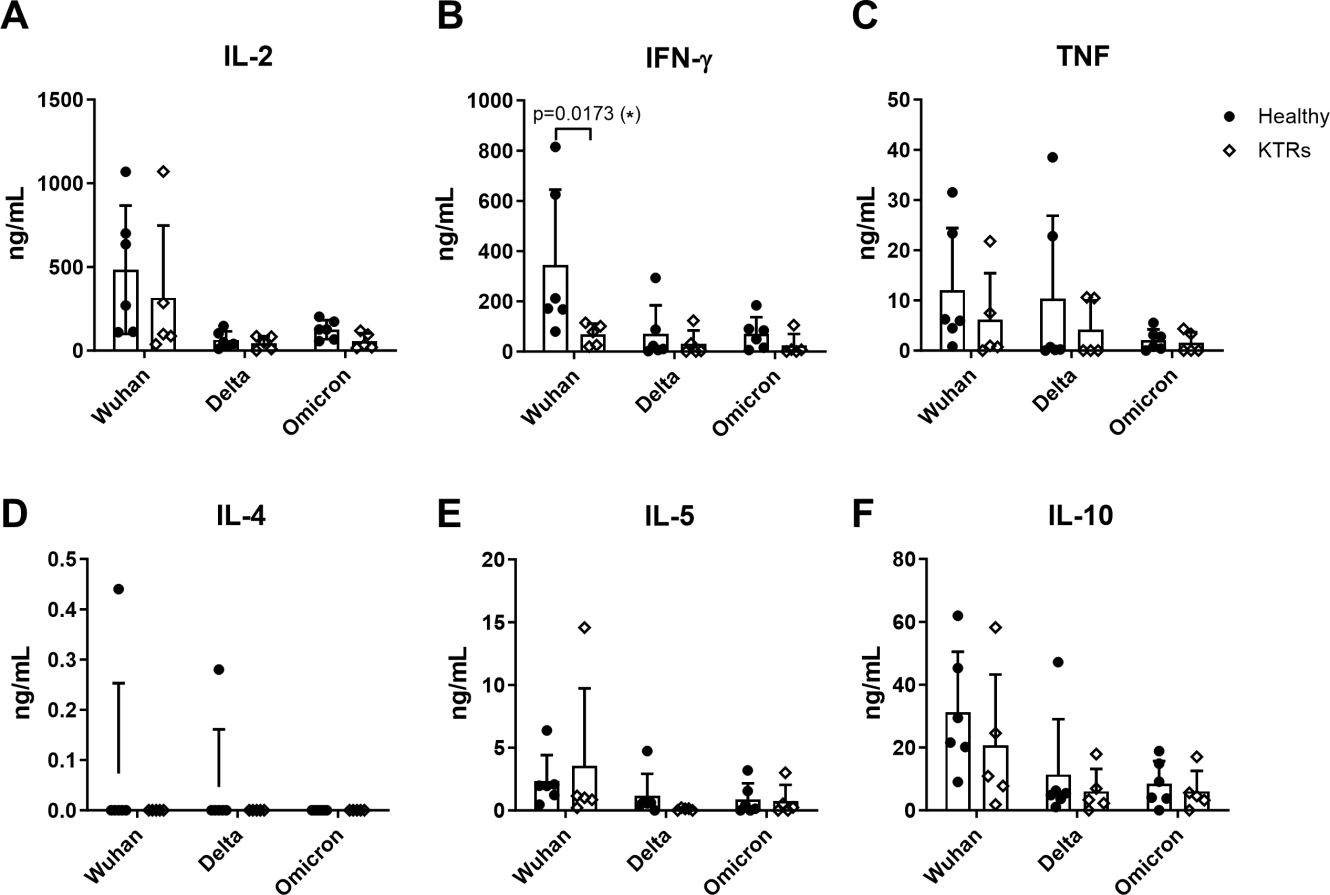
The secretion of IFN-γ of kidney transplant recipients (KTRs) was significantly lower than those of healthy donors after being stimulated with Wuhan spike peptide pools. (**A**) The cytokine levels of the cultured medium from PBMCs post-stimulation were compared within Wuhan, Delta, or Omicron spike peptide pool stimulation. The levels of Th1 cytokine, including (**A**) IL-2, (**B**) IFN-γ, and (**C**) TNF, were compared between the healthy donor and the KTR groups. The levels of Th2 cytokine, including (**D**) IL-4, (**E**) IL-5, and (**F**) IL-10, were compared between the healthy donor and the KTR groups. *P < 0.05.

Since we found no apparent differences in CD4 T cell cytotoxic reactivities between the healthy donor and the KTR groups using AIM assay, we aim to further determine the polarization of CD4 T cells by analyzing the secreted cytokine profiling of Th1 (TNF, IFN-γ, and IL-2) and Th2 (IL-4, IL-5, and IL-10) in response to spike peptide pool stimulation in PBMCs. In the results, Wuhan peptide pool stimulation induced higher levels of cytokines (IL-2, IFN-γ, TNF, IL-5, and IL-10) secretion in both KTRs and healthy donors when compared with Delta or Omicron BA.2 peptide pool stimulation, as shown in Fig. 3A through F. Among these cytokines, IL-2 and IFN-γ (Th1 cytokine) were the significant cytokines secreted in response to SARS-CoV-2 Wuhan, Delta, or Omicron BA.2 peptide pool stimulation (Fig. 3A and B). Despite observing no significant difference in T cell activation between KTRs and healthy donors, it was surprising to find that KTRs had lower secretion of Th1 cytokines, such as IL-2, IFN-γ, and TNF, compared to healthy donors (Fig. 3A through C). Importantly, we also found that the production of IFN-γ of KTRs was significantly lower compared to healthy donors as stimulated with the Wuhan peptide pools (Fig. 3B). These results suggested KTRs had more inadequate cellular responses triggered by vaccination than healthy donors.

## DISCUSSION

A previous study has shown that KTRs exhibit significantly lower humoral and cellular immunities against the Wuhan strain after the BNT162b2 prime boost compared to healthy individuals (12). In our experiment, we evaluated KTRs who were administered two to four doses of different vaccines. We found that the vaccines induced CD4 or CD8 T cell cytotoxicity responses against the ‘‘Wuhan’’ spike peptide stimulation without any significant differences compared to healthy donors. These results suggest that additional vaccine doses may improve T cell responses against the Wuhan strain in KTRs.

Given the ongoing mutation of SARS-CoV-2, the efficacy of administering a booster to KTRs against emerging variants is uncertain. For the healthy general population, recent reports have indicated that even individuals who have received two doses of vaccines designed based on the spike protein of the SARS-CoV-2 virus can still elicit T cell responses against Delta or Omicron, which is consistent with our data (14, 16). On the other hand, our results revealed that KTRs had impaired serological and cellular responses against the Omicron BA.2 variant. Specifically, in our study, KTRs exhibited a decrease in CD8 T cell response to Omicron BA.2 (Fig. 2F). Although the results suggested that KTR populations may have poor immune responses triggered by vaccination, we observed that two out of seven KTRs in our study were infected with Omicron BA.2 6 months and 1 year after receiving their last vaccination dose. Their symptoms were mild and did not require hospitalization. Upon review of the data of these two KTRs, we observed that, while they had almost no neutralizing antibodies against BA.2, they did exhibit T cell responses. These results indicated that, even if a vaccine does not induce a serological response, the cellular response can still play an important role. It also suggests that continuous booster shots may enhance the cellular immune response against the Omicron BA.2 variant in KTRs.

The KTR population has been facing a persistent problem of inadequate vaccine response. It is crucial to investigate the factors that may contribute to poor vaccine efficacy in KTRs. Numerous factors can affect vaccine response, including age, gender, timing and spacing of doses, vaccine type, previous exposure, genetics, health status, and environmental factors (19, 20). Factors associated with poor response to vaccination that are commonly discussed in the KTR population include older age, reduced GFR, lower lymphocyte count, diabetes, higher mycophenolic acid dose, belatacept use, treatment with Rituximab, and so on (21–26). Immunocompromised conditions resulting from administering immunosuppressive drugs to organ transplant recipients can also lead to defective antigen-presenting cell differentiation, maturation, and migration, directly affecting the magnitude and quality of B cell responses (27–30). A study has also shown that avoiding mycophenolic acid for a period can increase vaccine response (31). In light of these findings, we reviewed our study's medication records of KTRs. We found that individuals with low vaccine response presented higher levels of FK506 during the first month after vaccination. Thus, immunosuppressive drug use is an essential factor that affects KTRs' vaccine response.

While previous studies have assessed the T cell response against Wuhan in KTRs by measuring the intracellular cytokine of the T cells through AIM assay or IFN-γ ELISpot kit (12, 22, 32), we evaluated the level of extracellular cytokines. We found that KTRs had lower TNF, IFN-γ, and IL-2 secretions than healthy donors, regardless of being stimulated with Wuhan, Delta, or Omicron spike peptide pools. Notably, the secretion of IFN-γ was significantly decreased in KTRs compared to healthy donors when PBMCs were stimulated with Wuhan spike peptide pools. It has been known that IFN-γ can be produced by various cells, including T cells, NK cells, and dendritic cells. In T cells, IFN-γ is mainly produced by CD4 T cells and occasionally by CD8 T cells (33). IFN-γ not only helps CD8 T cells to kill infected cells but also acts as an adjuvant in many animal vaccine models to enhance vaccine-triggered T cell responses. Furthermore, a combination of factors, including immunosuppressive drugs, chronic inflammation, and rejection episodes, can contribute to the impaired secretion of IFN-γ by T cells in KTRs (34). However, a complete understanding of the mechanism underlying the decreased immune response induced by vaccines in the KTR population is still lacking, and further investigation is required to determine the underlying cause.

The limitations of this study include a low number of participants. Although there are discrepancies in the means of some data, the statistical analysis reveals no significant difference due to the small sample size. Moreover, our participants received different combinations of vaccine brands across three vaccine doses. To date, the research paper discussing the KTR population receiving mixed COVID-19 vaccine doses and the assessment of vaccine efficacy against the Omicron variant for individuals who have not been infected was absent. Therefore, this study can serve as a prospective experiment to provide a reference for future research.

These results revealed that KTRs have weaker immunity against SARS-CoV-2 triggered by vaccination than healthy individuals. To address this issue, there are effective measures available to reduce the mortality rate of KTRs, including administering SARS-CoV-2-neutralizing monoclonal antibody combination, such as tixagevimab and cilgavimab (35, 36) or receiving a next-generation vaccine to provide additional protection against the Omicron variant (37). Most importantly, different strategies to enhance KTR immunity triggered by vaccines are needed.

## SUPPLEMENTAL MATERIAL

Supplemental figures spectrum.00004-24-s0001.docx Fig. S1-S5.

ASM does not own the copyrights to Supplemental Material that may be linked to, or accessed through, an article. The authors have granted ASM a non-exclusive, world-wide license to publish the Supplemental Material files. Please contact the corresponding author directly for reuse.
