# Supplementary material for: Optimizing SARS-CoV-2 vaccine responses in kidney transplant recipients: an urgent need
Source: Microbiol Spectr. 2024 May 15;12(6):e00004-24. doi: 10.1128/spectrum.00004-24 (PMC11237705; doi:10.1128/spectrum.00004-24)
Supplement: Supplemental figures — Fig. S1-S5. [file spectrum.00004-24-s0001.docx]

**Supplementary Figure 1 | The levels of anti-nucleocapsid antibodies were measured.**

**Supplementary Figure 2 | Characteristics of donor cohorts**

Detailed information about each donor was provided, including the vaccine brand, the receiving date of each dose, the duration after the last vaccination, and the duration after the kidney transplant. (AZD1222 (Oxford-AstraZeneca, AZ), mRNA-1273 (Moderna), BNT162-b2 (Pfizer–BioNTech, BNT), and MVC-COV1901(Medigen Vaccine Biologics Corporation, MVC))


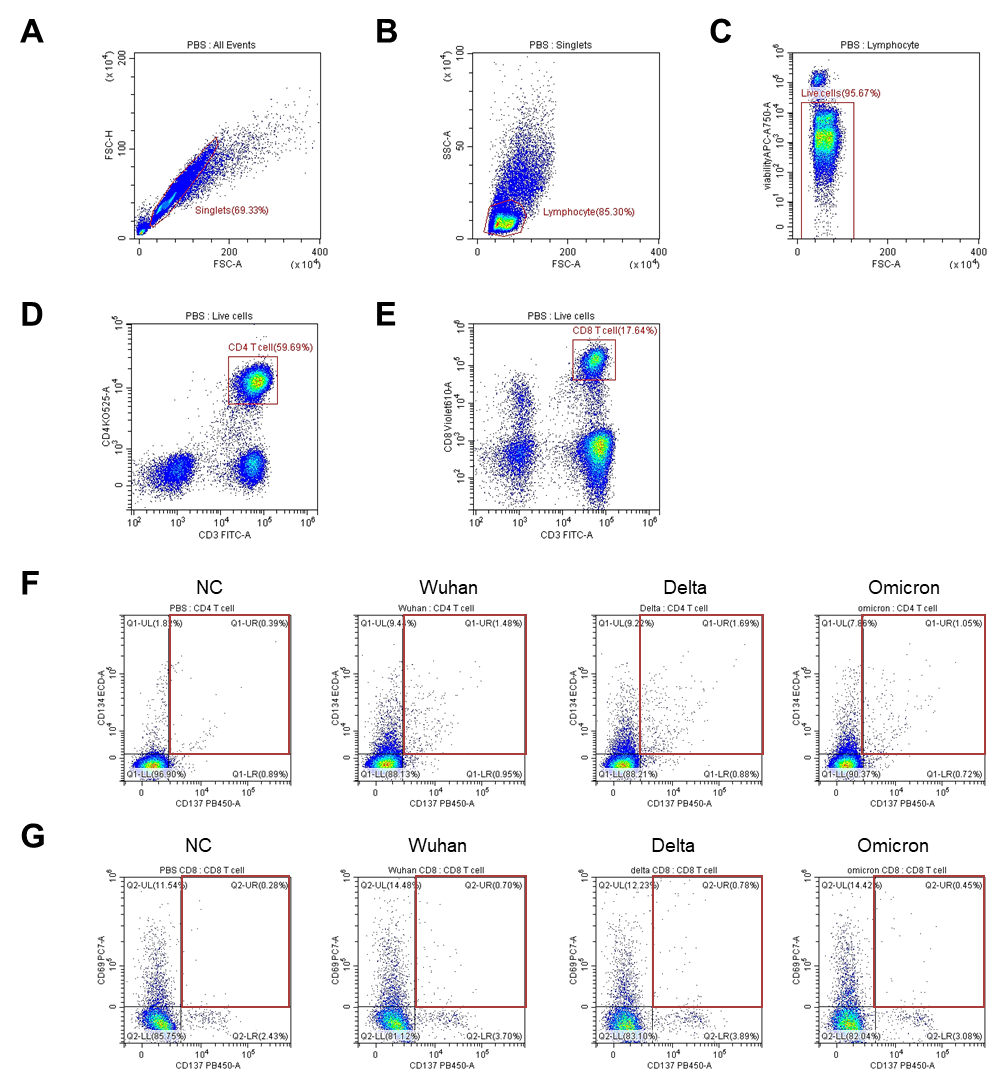


**Supplementary Figure 3 | Flow cytometry gating strategy for AIM assay. (A)** Firstly, singlets were selected based on FSC-A vs. FSC-H **(B), and** then the lymphocytes were gated on FSC vs. SSC. **(C)** Subsequently, live cells were gated, and the frequency of **(D)** CD4 T cells (CD3+CD4+) and **(E)** CD8 T cells (CD3+CD8+) from the PBMCs of recipients were selected. Antigen-specific T cells will be measured as a percentage of **(F)** AIM-positive CD4 T cells (CD134 and CD137 co-expression) and **(G)** AIM-positive CD8 T cells (CD69 and CD137 co-expression) after stimulation of PBMCs by different spike peptide pools. AIM-positive gates were drawn relative to the condition of negative PBS control (NC) for each donor, and the data were shown after being subtracted against the negative PBS control.


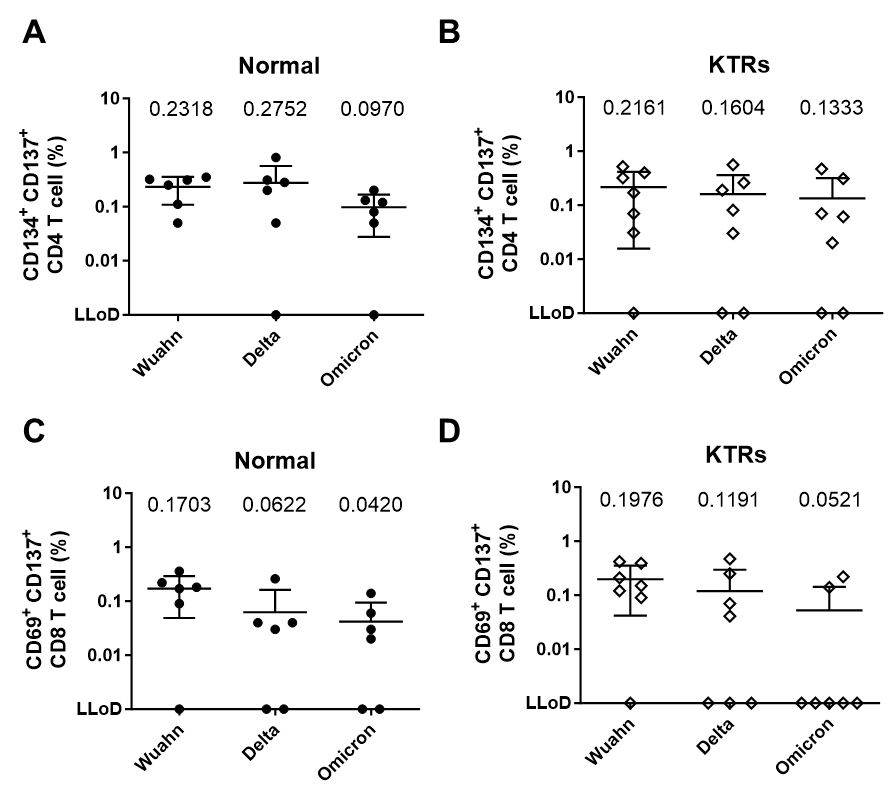


**Supplementary Figure 4 | Comparison of T cells reactivity in response to different SARS-CoV-2 spike peptide pools.** SARS-CoV-2-specific CD4+ T cells reactivity in response to Wuhan, Delta, and Omicron were compared within (A) the healthy donors’ group or (B) the KTRs group. SARS-CoV-2-specific CD8+ T cells reactivity in response to Wuhan, Delta, and Omicron were compared within (C) the healthy donors’ group or (D) the KTRs group.


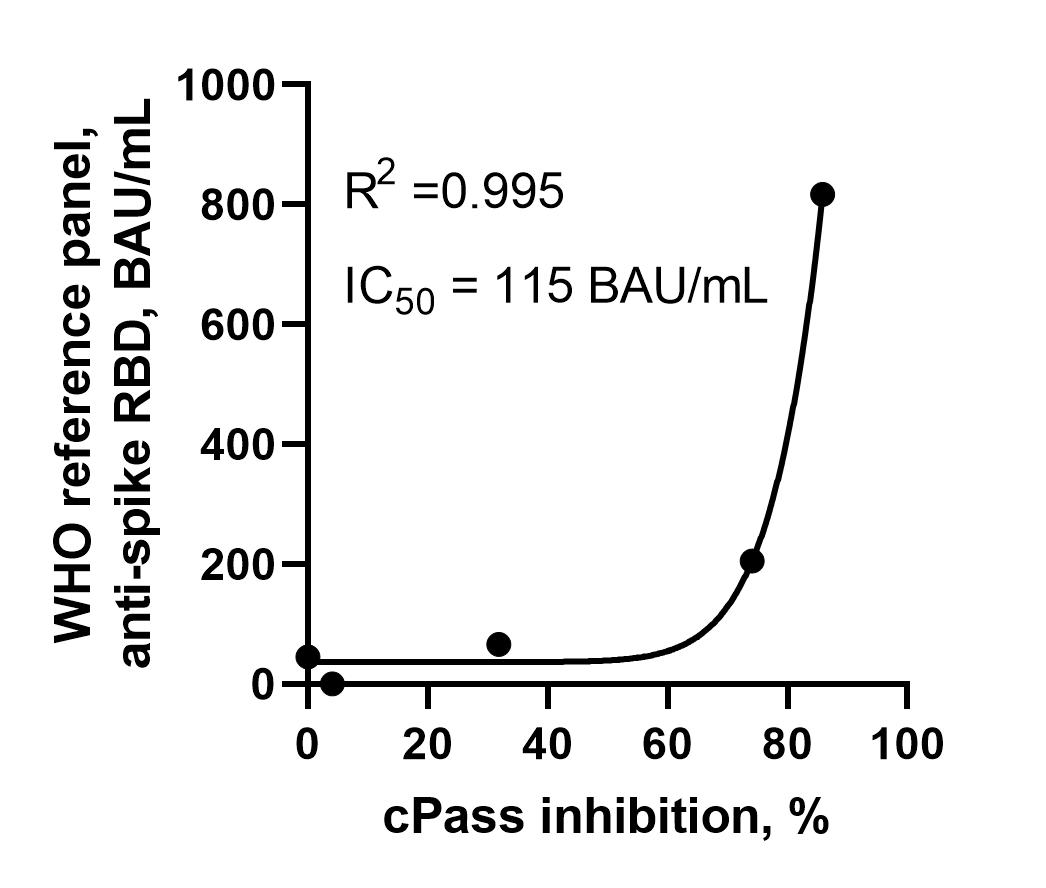


**Supplementary Figure 5 |** **The correlation between cPASS inhibition values (%) and anti-RBD levels (BAU/mL, using the WHO reference panel) (WHO: World Health Organization)**
